# Inhibition characteristics research of aluminum alloy polishing dust explosion through addition of ultrafine Al(OH)_3_ inerting agent

**DOI:** 10.1016/j.heliyon.2023.e19747

**Published:** 2023-09-01

**Authors:** Chen Lv, Xinqun Wang, Sheng Xue, Xinxing Xia, Shuang Wang

**Affiliations:** a College of Quality and Safety Engineering, China Jiliang University, Hangzhou, 310018, China; b Key Laboratory of Safety and High-efficiency Coal Mining, the Ministry of Education(Anhui University of Science and Technology), Huainan, 232001, China

**Keywords:** Safety science, Ultrafine Al(OH)_3_ powder, Aluminum alloy polishing dust, Dust explosion

## Abstract

Investigations into the deactivation of explosion sensitivity and reduction of flame propagation for aluminium alloy polishing wastes were carried out by the addition of ultrafine Al(OH)_3_ inerting agent. Meanwhile, high-purity aluminium powders with similar mean diameters were also used as a comparative study. The explosion propagation characteristics of high-purity aluminium dust and aluminium alloy polishing waste dust under different inerting ratios (ε) were tested and investigated using a standardised Hartmann tester and a developed experimental platform. The results show that the minimum ignition energy of high-purity aluminium powder is between 40 and 45 mJ, and the minimum ignition energy of aluminium alloy polishing waste is between 500 and 550 mJ, which is one order of magnitude higher than that of high-purity aluminium powder. The lower explosion limit concentration of aluminium alloy polishing waste dust is 150 g/m^3^, which is 53.33% of that of high-purity aluminium powder. According to the analysis of the SEM image, the main reason is that the spherical particles of high-purity aluminium dust have a folded surface and good dispersion. Compared with the smooth fibre surface of aluminium alloy polishing waste dust, the former is easier to contact with air and the contact area is larger. Therefore, in engineering practice, it is not appropriate to use high-purity aluminium dust-related explosion parameters as the basis for the risk assessment of combustion and explosion at aluminium alloy polishing work sites. In addition, as the dust concentration decreases, the combustion intensity of high-purity aluminium dust and aluminium alloy polishing waste dust also decreases, and the flame propagation appears to be a discontinuous phenomenon. The peak flame propagation velocity of aluminium alloy polishing waste is 7.368 m/s at a concentration of 300 g/m^3^, which is 56.85% of that of high-purity aluminium powder. As the inerting ratio increases, the propagation velocity of the explosion flame slows down. When the inerting ratio reaches 30%, the minimum ignition energy of aluminium alloy polishing waste is inerted to 1 J, and self-sustained flame propagation cannot be formed. The results show that the ultra-fine Al(OH)_3_ powder has a significant inerting effect and is a realistic possibility in the production of aluminium alloy polishing.

## 1 Introduction

Relevant literature research shows that, as an important part of the surface treatment of aluminium alloy die-cast blanks, in the production process of aluminium products grinding, polishing, blasting and other processing processes, the explosion sensitivity and intensity of various types of metal aluminium dust wastes generated are significantly higher than those of organic dusts [[1], [2], [3], [4], [5]]. Therefore, dust combustion and explosion at the aluminium product processing site is a major safety accident hazard, must take scientific and effective dust explosion prevention and control measures to prevent the occurrence of explosive accidents, once the dust explosion prevention and control measures taken are unreasonable, it will inevitably lead to dust combustion. Explosion accident [6]. For example, in 2014, the “8.2″ dust explosion accident occurred in an aluminium alloy polishing workshop in Kunshan, Jiangsu Province, causing a total of 97 deaths and 163 injuries. In 2016, an aluminium dust explosion accident occurred in a hardware processing factory in Shenzhen, Guangdong, causing a total of 5 deaths and 5 injuries. How to effectively prevent and control the occurrence of such accidents is currently the focus of safety research at home and abroad. In 2016, Hu [7] and Marmoa [8] laid the foundation for exploring the flame structure and propagation law of aluminium powders with different particle sizes. Taking high-purity aluminium dust as the research object, a relationship model between the flame propagation speed, maximum flame temperature and dust particle size of aluminium powders was established. In 2017, based on the 20 L explosive ball device, Li [9] systematically studied the explosion characteristics of 6 kinds of micron-sized aluminium powders with different particle sizes and established the functional relationship between dust particle size and explosion overpressure. In 2019, Chen [10] clarified the influence of dust particle size distribution, specific surface area, dust concentration, etc. On the explosion characteristic parameters through the explosion experiments of ultra-fine aluminium powders under different experimental conditions. In 2019 and 2021, Pranav [11] and José [12] took ultra-fine aluminium powder as the research object and revealed the influence of dust particle size distribution, surface morphology, initial turbulence and other factors on the explosion characteristic parameters and flame propagation process. It is proposed that only when the oxide layer on the dust surface is melted under a high-temperature ignition source can the fire phenomenon occur, which provides a theoretical basis for assessing the risk of dust explosions. However, compared with the aforementioned aluminium powder, the polishing and blasting waste generated during the production process is mostly alloy dust and accompanied by other impurities, which cannot simply follow the explosion parameters of the former, and there are significant differences in the actual explosion hazard for different production sites and processes. In 2018, Li [13] studied the explosion characteristics of aluminium alloy polishing dust and the applicability of the test equipment by using the improved Hartmann test equipment and classified the explosion characteristics of aluminium alloy polishing dust into three categories: easily explosive, explosive and non-explosive. In addition, taking appropriate explosion prevention and control measures based on the actual production site is another key issue that the industry has always paid attention to. By adding inert powder to the combustible dust, it can help to reduce its ignition sensitivity and explosion intensity. In 2016 and 2017, Luo [14,15] found that the inert powder mixed with combustible dust, on the one hand, can be pyrolysed to absorb a large amount of heat, reduce the temperature of the explosive reaction zone and prevent the explosive chain reaction, and on the other hand, its pyrolysis products can capture the free radicals in the explosive reaction and inhibit the explosive reaction chain. Addai [16] investigated the effect on ignition performance by applying (NH_4_)_2_SO_4_ inert powder to corn starch, the experimental results showed that when the mass fraction of (NH_4_)_2_SO_4_ was 60%, the minimum ignition energy of corn starch could be improved, inerted to 1 J, losing the ability to propagate explosions. Amyotte [17] comprehensively investigated the effects of CaCO_3_, NH_4_H_2_PO_4_ and NaHCO_3_ powders on the explosion resistance of Pittsburgh mine coal dust, corn starch and aluminium powder, and the experimental results showed that in the mixed system of combustible dust/inerting powder, only when the mass fraction of the inerting agent is higher than that of the combustible dust, the explosion protection effect of inerting can be manifested. However, at production sites, the potential impact on the subsequent use of the product caused by the use of combustible dust explosion prevention and control through the application of inerting powder when the powdered material is used as a final product is an issue that needs to be considered in production practice.

In summary, considering the actual production site, aluminium product processing mainly produces aluminium alloy polishing dust, which is quite different from the combustion and explosion propagation characteristics of pure aluminium dust, therefore the dust explosion risk assessment process at the aluminium product processing site should not simply follow the relevant international standards to develop explosion prevention and control measures. For this reason, the authors of this paper take a typical aluminium alloy polishing dust as the object of study and examine the effect on explosion sensitivity by applying a certain amount of ultrafine Al(OH)_3_ powder and exploring its inerting effect on explosion flame propagation, in order to provide the necessary scientific basis for the rational formulation of dust combustion and explosion prevention and control measures. Moreover, while dry dust removal is frequently used in the manufacture of aluminum alloy products, a lack of suitable prevention and control procedures can easily result in dust explosion disasters. A such example is the “8.2″ dust explosion catastrophe in Kunshan (2014). Since then, wet dust removal has been employed in various instances; nevertheless, the interaction of aluminum alloy dust and water may easily generate hydrogen gas, which can offer new threats in specific conditions, and the disposal slurry and waste water can also cause secondary environmental issues. In this circumstance, this article aimed to eliminate or reduce the harmfulness of explosions by adding an inerting chemical to disposal waste dust. It may have potential practical applications.

## 2 Experimental test system establishment

### 2.1 Selection and preparation of experimental samples

**Fig. 1:**
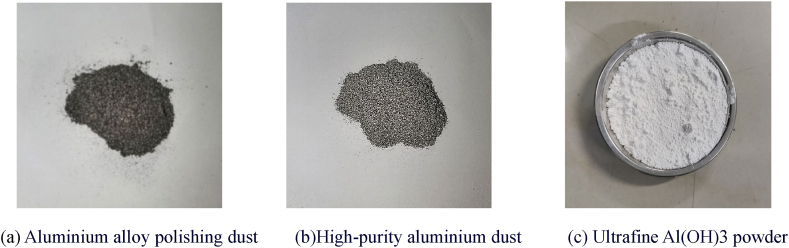
Experimental samples.

The aluminium alloy polishing dust (Fig. 1(a)) is selected from the dust (4032 silicon-aluminium alloy) generated during the belt grinding process at an aluminium product processing factory in Sanmen City, Zhejiang Province. The dust collected on site is first placed in a laboratory vacuum drying box and dried at 40 °C for 12 h, and then the dried aluminium alloy polishing dust is removed and processed and sieved using a QM-3SP4 planetary ball mill to obtain experimental samples with particle size less than 75 μm. For comparative analysis, high-purity aluminium dust (Fig. 1(b)) of the same size with 99.5% aluminium content was selected for the contrast study of explosion parameters and the inerting explosion protection effect.

Ultrafine Al(OH)_3_ powder (Fig. 1(c)) is selected as the inerting agent, which is in a heated state and will undergo the chemical decomposition reaction, it can absorb a lot of heat during the pyrolysis process and reduce the temperature of the explosion reaction zone, reduce the ignition sensitivity of combustible dust, while its pyrolysis products can also take away the free radicals in the explosive reaction, inhibit the progress of the explosion reaction chain and reduce the explosion power. On the other hand, the mixture of Al(OH)_3_ ultrafine powder and aluminium alloy polishing dust can also be used as a flocculant in the sewage treatment process, so that the waste generated in the production can be recycled and reused to achieve the purpose of high efficiency, environmental protection and energy saving. In the laboratory, the experimental samples of Al(OH)_3_ ultrafine dust with a particle size of less than 6.5 μm were prepared by drying, processing and sieving in the same way as above.

### 2.2 Experimental test device and steps

**Fig. 2:**
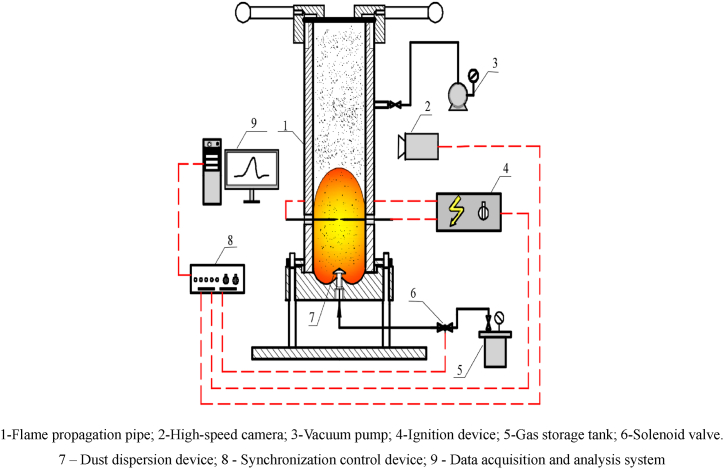
Experimental test system of explosive flame propagation.

The effect on the minimum ignition energy and lower explosion limit concentration of aluminium alloy polishing dust was investigated by applying an ultrafine Al(OH)_3_ inerting agent using the standard Hartmann explosion apparatus, following the appropriate test procedure and determination rules. The explosion flame propagation process was investigated using a self-developed explosion tube test apparatus, as shown in Fig. 2, the experimental apparatus mainly consists of a flame propagation tube, dust dispersing device, an ignition device, a high-speed camera, a synchronous controller, and a data acquisition and analysis system, where the main body of the explosion apparatus is a stainless steel flame propagation tube with a height of 500 mm and a cross-section of 100mm × 100 mm, which is embedded on both sides of the observation window with a scale to record the flame propagation process. The ignition electrode is located 100 mm from the bottom of the tube and the dust cloud is ignited by an electric spark, the ignition energy is set at 20 J. The top of the tester is sealed with a 0.1 mm thick polyethene pressure relief film to ensure safety during the test. The test dust was placed in a container at the bottom centre of the pipe and blown through a mushroom-shaped nozzle using compressed air at an initial pressure of 0.7 MPa to roll it up and form a uniformly distributed cloud of dust in the test pipe.

Experimental steps: 1) According to the set inerting ratio as shown in equation (1), the test samples of aluminium alloy polishing waste dust mixed with Al(OH)_3_ ultrafine dust and the same particle size of high-purity aluminium dust mixed with Al(OH)_3_ ultrafine dust were configured separately. 2) According to the experimental requirements, a certain amount of experimental sample is put into the container at the bottom centre of the explosion pipeline. 3) Start the vacuum pump to pre-vacuum the pipeline. 4) Adjust the air pressure to 0.7 MPa. 5) Start the test control system, issue a command to open the nozzle solenoid valve to form a dust cloud, trigger the ignition device after a delay of 150 ms, and simultaneously trigger the high-speed camera and the experimental data acquisition system.

(1) $$\varepsilon =\frac{m_{1}}{m_{2}}$$

in equation (1), the ε is the inerting ratio, $m_{1}$ is the quality of ultrafine Al(OH)_3_ powder, $m_{2}$ is the combustible dust quality.

### 2.3 Morphology analysis of experimental samples

**Fig. 3:**
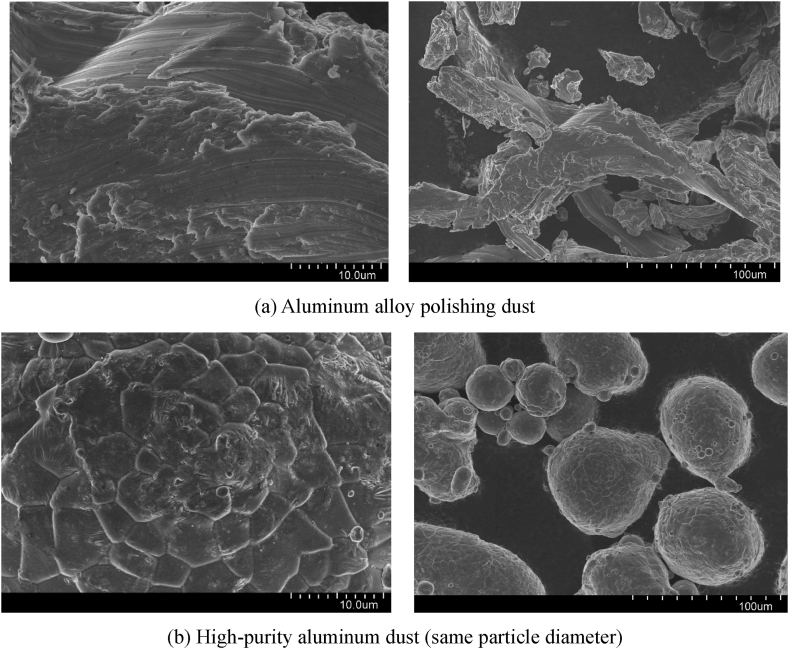
SEM images of the experimental samples observed by scanning electron microscopy.

After the test specimens were prepared according to the experimental requirements, the surface microscopic morphology of the test specimens was observed using the FEI Nova NanoSEM 450 scanning electron microscope, and then the particle size distribution of the test specimens was tested using the Malvern Mastersizer 3000 Malvern particle size analyser, and the physical properties of the test specimens were further analyzed. The specific analytical results are as follows:

Aluminium polishing waste dust and high-purity aluminium dust have been tested using a Malvern particle size analyser and have approximately the same mean particle diameter, D_50_ = 49.78μm. The SEM image of the experimental sample of aluminium polishing waste dust is shown in Fig. 3(a). It can be seen from the SEM image that the aluminium polishing waste dust particles are smooth and fibrous in shape, with an obvious fibrous structure and a small amount of polishing paste adhered to the surface and interspersed with fine particles, which are easy to adhere when mixed with ultrafine Al(OH)_3_ inerting agent. The SEM image of the experimental sample of high-purity aluminium, as shown in Fig. 3(b), it can be seen from the SEM image that the dust particles are spherical with a folded surface and are well dispersed, which are not easy to adhere when mixed with ultrafine Al(OH)_3_ inerting agent.

**Fig. 4:**
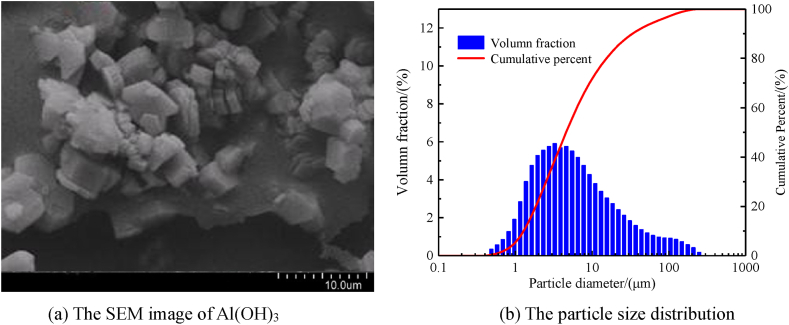
The particle size distribution of ultrafine Al(OH)_3_ powder and SEM image.

The experimental samples of Al(OH)_3_ ultrafine dust, the surface morphology and particle size distribution of the dust particles are shown in Fig. 4(a) and (b), the average particle diameter of Al(OH)_3_ ultrafine is 6.5 μm, there is a degree of agglomeration of small dust particles. During the experiment, the ultrafine Al(OH)_3_ inerting agent and aluminium alloy polishing dust were homogeneously mixed by a three-dimensional mixer according to the set inerting ratio.

## 3 Result and discussion

### 3.1 Passivation effect of inerting agent on dust explosion sensitivity

#### 3.1.1 Effect of inerting agent on the minimum ignition energy of dust

The minimum ignition energy is the minimum spark energy that will ignite the dust and sustain combustion. Starting with a spark of an energy value that reliably ignites the dust cloud for a given dust mass concentration, the spark energy value is gradually reduced until no ignition occurs. During the experiment, a certain amount of high-purity aluminium dust and aluminium alloy polishing dust experimental samples were weighed, respectively, and the initial mass concentrations of high-purity aluminium dust and aluminium alloy polishing dust in the Hartmann tube were both set to 400 g/m^3^, and the ignition energy and mass concentration were alternately changed for testing, and finally the minimum ignition energy of these two kinds of dust was measured, the test results are shown in Table 1 and Table 2. (Note that T in the table means that the test dust is ignited and F means that the test dust is not ignited).

**Table 1:** Minimum ignition energy test results of high-purity aluminum dust.

Ignition energy (mJ)	70	65	60	55	50	45	40	35	30
High-purity aluminum dust	T	T	T	T	T	T	F	F	F

**Table 2:** Minimum ignition energy test results of aluminum alloy polishing dust.

Ignition energy (mJ)	900	850	800	750	700	650	600	550	500	450
Aluminum alloy polishing dust	T	T	T	T	T	T	T	T	F	F

According to the test results in Table 1, Table 2, the minimum ignition energy range of high-purity aluminium dust is between 40 and 45 mJ, the minimum ignition energy of aluminium alloy polishing waste dust is between 500 and 550 mJ, the minimum ignition energy of high-purity aluminium dust is only about 8.1% of aluminium alloy polishing waste dust. The main reason for this is that the spherical particles of high-purity aluminium dust with wrinkled surfaces and good dispersion are more accessible to air and have a larger contact surface than the smooth fibrous particle surfaces of aluminium alloy polishing waste dust. At the same time, the test results also show that the explosion sensitivity of high-purity aluminium dust is much higher than that of aluminium alloy polishing dust, so it is not appropriate to simply follow the explosion sensitivity index of aluminium dust for the explosion risk assessment of aluminium alloy grinding and polishing processes.

**Fig. 5:**
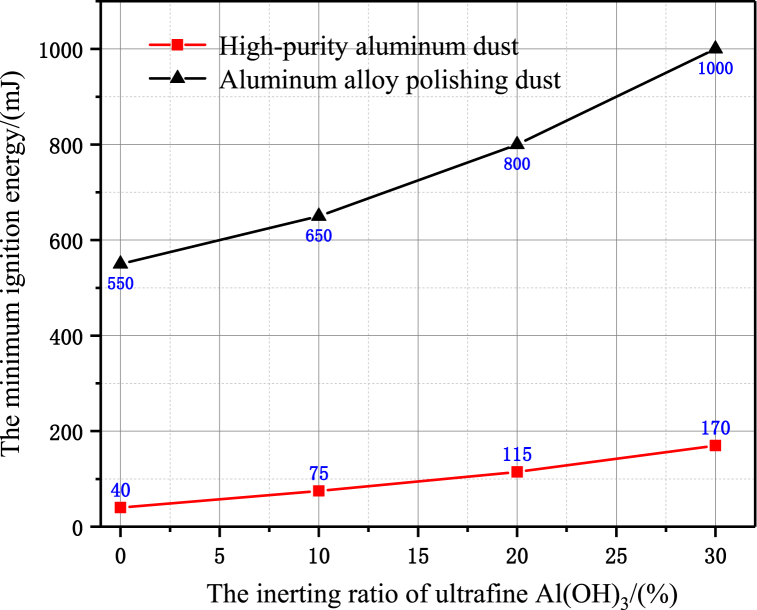
Effect of ultrafine Al(OH)_3_ powder on minimum ignition energy.

The effect of the use of ultrafine Al(OH)_3_ inerting agent on the minimum ignition energy also varies. The results of the experimental tests are shown in Fig. 5. When the inerting ratio is 10%, the minimum ignition energy of high-purity aluminium dust is inerted from 40 mJ to 75 mJ, and the minimum ignition energy of aluminium alloy polishing waste dust is inerted from 550 mJ to 650 mJ. When the inerting ratio is 30%, the minimum ignition energy of high-purity aluminium dust is inerted from 40 mJ to 170 mJ, and the minimum ignition energy of aluminium alloy polishing waste dust is inerted from 550 mJ to 1 J, which belongs to the difficult ignition level. This shows that the inerting effect of ultrafine Al(OH)_3_ inerting agent on the ignition sensitivity of aluminium alloy polishing dust is more significant.

#### 3.1.2 Effect of inerting agent on the lower limit concentration of dust explosion

The lower explosive limit concentration is the lowest concentration at which a dust cloud can undergo self-sustained combustion for a given ignition energy. In the IEC/FDIS 80079-20-2 specification, the criterion for determining whether the dust explodes is given as where is the maximum overpressure generated by a dust explosion and is the initial overpressure generated by an igniter explosion without the involvement of combustible dust. In this experiment, the initial mass concentration of the test dust was set at 200 g/m^3^. If an explosion occurred during the test, the dust concentration was reduced by 10 g/m^3^ to continue the test; if no explosion occurred during the test, the experiment was repeated three times continuously at this concentration. The maximum dust concentration at which no explosion occurs three consecutive times is the lower limit of the test dust explosion concentration. The test results are given in Table 3.

**Table 3:** Test results of dust explosion lower limit concentration.

Experimental samples	Dust concentration/(g·m^−3^)									
	70	80	90	100	110	120	130	140	150	160
High-purity aluminum dust	F	T	T	T	T	T	T	T	T	T
Aluminum alloy polishing dust	F	F	F	F	F	F	F	F	T	T

Based on the experimental results in Tables 3 and it can be seen that the lower explosion limit concentration of high-purity aluminium dust is 80 g/m^3^ and the lower explosion limit concentration of aluminium alloy polishing dust is 150 g/m^3^, the former lower explosion limit concentration is only 53.3% of the latter, the combustion reaction activity is higher, it can be seen that the explosion sensitivity of high-purity aluminium dust is much higher than that of aluminium alloy polishing dust, and the explosion risk is also greater.

**Fig. 6:**
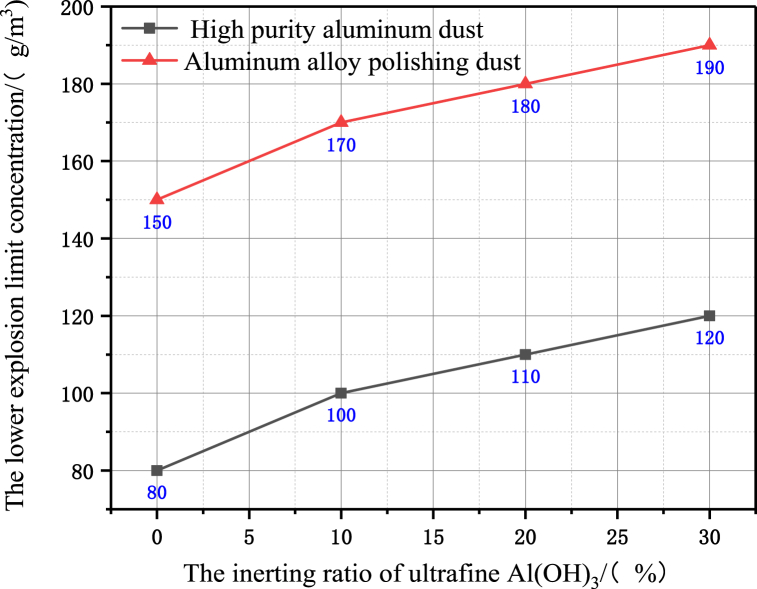
Effect of ultrafine Al(OH)_3_ powder on lower explosion limit concentration.

From the results in Fig. 6, the effect of different amounts of Al(OH)_3_ ultrafine inerting agent on the lower limit of combustible dust explosion concentration, when the inerting ratio is 10%, the lower explosion limit concentration of high-purity aluminium dust is inerted from 80 g/m^3^ to 100 g/m^3^, and the lower explosion limit concentration of aluminium alloy polishing dust is inerted from 150 g/m^3^ to 170 g/m^3^. When the inerting ratio is 30%, the lower explosion limit concentration of high-purity aluminium dust is inerted from 80 g/m^3^ to 120 g/m^3^, and the lower explosion limit concentration of aluminium alloy polishing dust is inerted from 150 g/m^3^ to 190 g/m^3^. Under the same inerting ratio, the inerting effect of the lower explosion limit of aluminium alloy polishing waste dust is significantly better than that of high-purity aluminium dust.

### 3.2 Explosion-proof effect of ultrafine Al(OH)_3_ inerting agent on aluminum alloy polishing waste dust

#### 3.2.1 Experimental study of the flame propagation characteristics of dust explosions

**Fig. 7:**
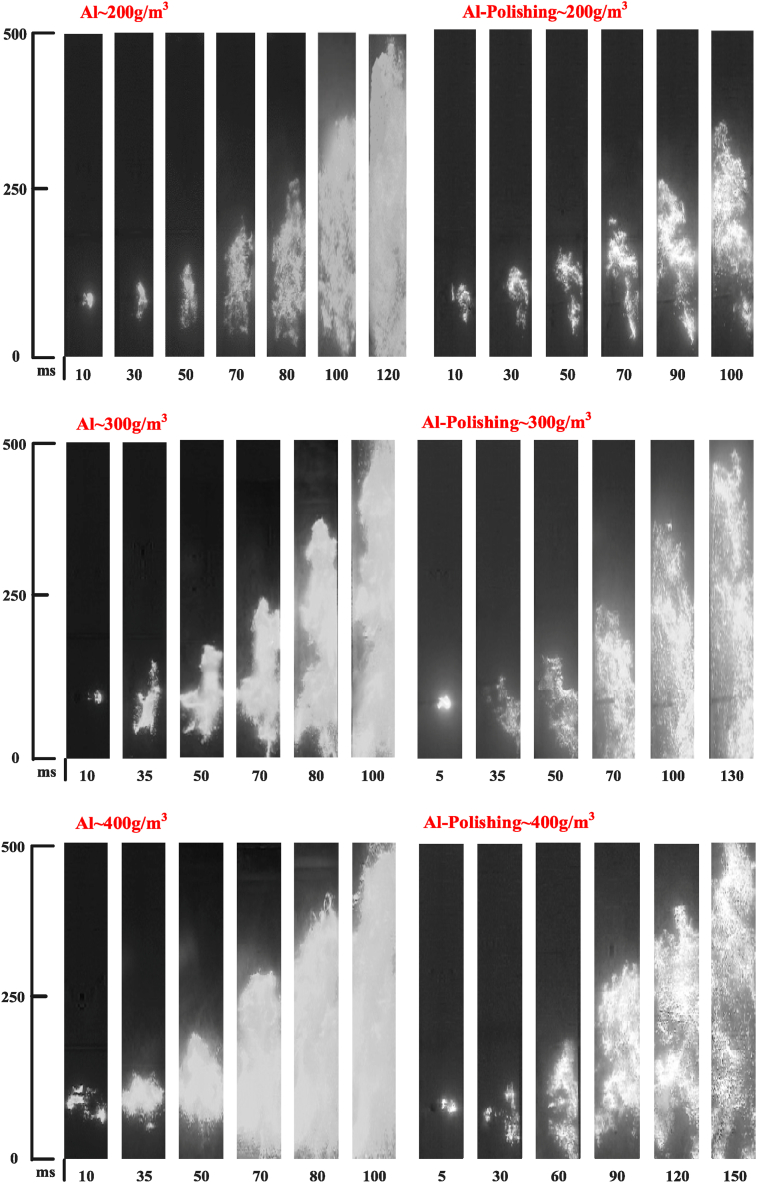
Comparison of explosion flame structure under different dust concentration fields.

Based on the above experimental results of the explosion lower limit concentration, a certain amount of high-purity aluminium dust and aluminium alloy polishing dust experimental samples were respectively selected and configured in the Hartmann tube with different mass concentration fields of 200 g/m^3^, 300 g/m^3^ and 400 g/m^3^ to carry out the explosion flame propagation characteristics test, the comparison of the explosion flame structure at different dust mass concentrations is shown in Fig. 7.

The experimental results show that with the continuous reduction of the experimental dust concentration, the explosion flame combustion intensity of high-purity aluminium dust and aluminium alloy polishing waste dust also decreases, and flame propagation will appear discontinuity phenomenon. Conversely, with the increase of mass concentration, high-purity aluminium powder explosion flame intensity increases, flame propagation speed increases, flame propagation continuity becomes better, aluminium alloy polishing waste dust, although flame brightness has increased, compared to high-purity aluminium powder explosion flame is weaker, explosion pressure is smaller, flame propagation speed is slower, there are still a large number of luminous point flame near the flame front, flame structure continuity is poor. The main reason for this result is that aluminium alloy polishing waste dust contains non-combustible impurities and the dust particle structure is highly fibrous, so the particles near the electrodes cannot fully contact with oxygen and decompose in the initial process of the explosion, so the flame is more discrete, discontinuous, and the number of luminous point flames around the core of the flame is higher.

To ensure the intensity of the explosion flame propagation and at the same time to facilitate clear filming by high-speed cameras, the effect of the application of ultrafine Al(OH)_3_ powder on the explosion flame propagation process was investigated under the experimental condition of a combustible dust mass concentration of 300 g/m^3^. Flame propagation images taken by a high-speed camera were recorded at specific time steps.

**Fig. 8:**
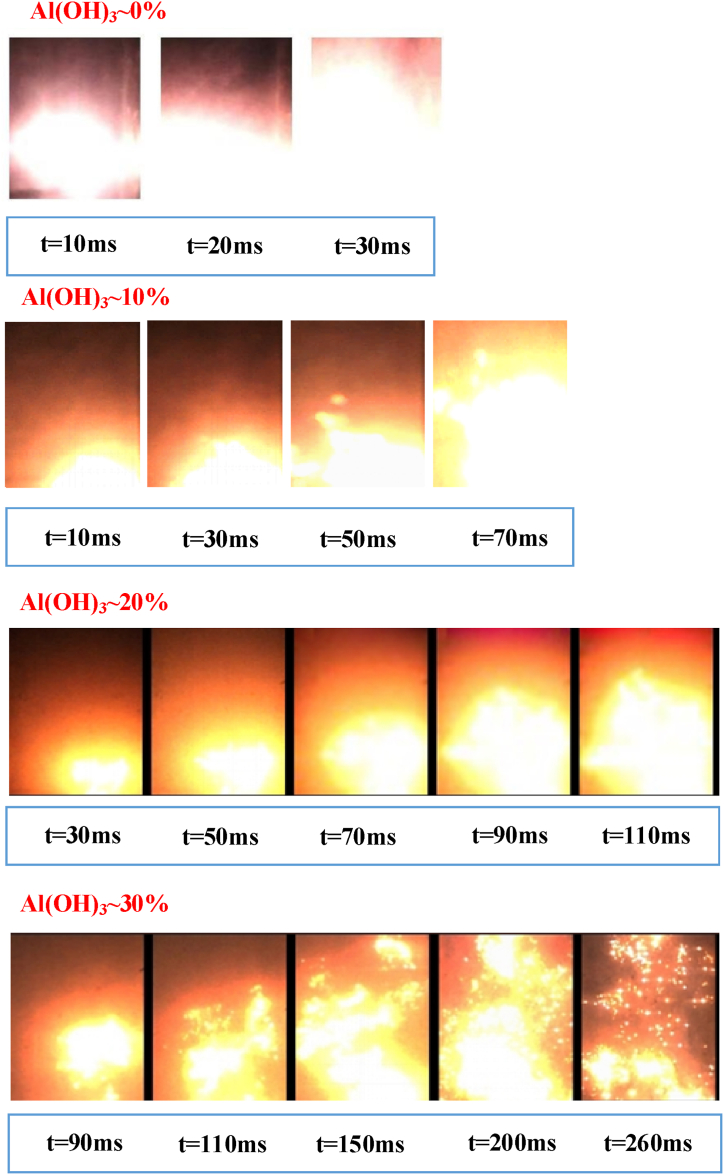
Variation of the flame shape of aluminum powder/air mixture under different amounts of inerting agent.

Starting from the ignition electrode, the shape of the explosion flame 200 mm above it is shown in Fig. 8. When no Al(OH)_3_ ultrafine powder is applied, the flame propagation tends to accelerate significantly at 10 ms, accompanied by more intense flame radiation. The combustible dust mass concentration remains unchanged, and the overall propagation of the explosion is slowed by the application of 10% ultrafine Al(OH)_3_ powder, with the flame front reaching the side wall of the tube at approximately 50 ms, despite the irregular shape of the flame front, the overall structure of the flame is relatively intact, with a significant acceleration trend at approximately 70 ms. This shows that the application of 10% ultrafine Al(OH)_3_ powder has a more limited inerting effect on the explosion propagation of high-purity aluminium powder.

When the inerting ratio is increased to 20%, the Al(OH)_3_ ultrafine powder shows heat absorption efficiency, the radiation intensity of the explosion flame decreases and an intermittent flame zone appears during the flame propagation process. As the amount of Al(OH)_3_ ultrafine powder applied increases, the flame dispersion increases and the flame colour gradually changes to light red.

**Fig. 9:**
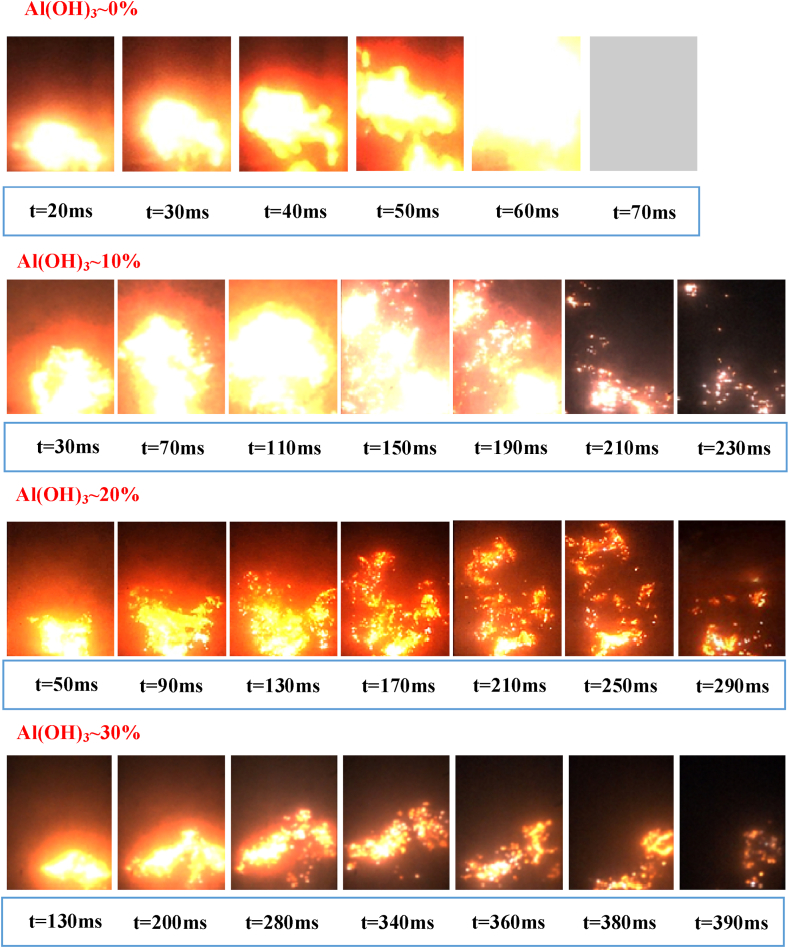
Variation of the flame shape of aluminum alloy polishing associated dust/air mixture under different amounts of inerting agent.

The effect of the addition of Al(OH)_3_ ultrafine powder on the explosive flame morphology of aluminium alloy polishing waste dust is shown in Fig. 9. When no Al(OH)_3_ ultrafine powder is added, the initial flame radiation intensity of the polishing dust is significantly attenuated compared to that of higher-purity aluminium powder. The small dust particles near the electrode absorb energy through thermal convection and conduction, and the surface temperature of the particles increases significantly and begins to melt, and the combustion zone is formed after the decomposition of gaseous combustibles and oxygen are fully mixed. Flame radiation is most intense in the combustion zone. There is a small amount of non-combustible impurities in the polishing waste dust, which affects the endothermic melting of the dust particles in the preheating zone, and the flame radiation in this zone is relatively weak. In addition, the particles that do not participate in the reaction, or only partially react, are suspended in the unburned area at the top of the tube, and the flame radiation is relatively weak. Before 50 ms, the outline of the combustion zone, preheating zone and unburned zone is clear, and the relatively strong flame radiation begins to appear at 60 ms.

When the inerting ratio is increased to 10%, the inerting effect on flame propagation is more obvious. At the initial ignition stage, the explosion flame's acceleration trend is obviously slower. By comparing the flame propagation height at different times, the application of an inerting agent significantly delays the longitudinal flame propagation. With the increase of time, the cooling effect of Al(OH)_3_ ultrafine powder damages the continuity of the flame structure, and a large extinction area appears in the combustion pellets, and the explosion flame is no longer smooth and continuous, but shows a discrete state. By increasing the inerting ratio to 20%, the above inerting effect is more fully played, the instability of the explosion propagation is increased, the combustion zone is more discrete, and the flame velocity shows an oscillating state. If the inerting ratio is increased to 30%, the flame may continue to propagate in the initial stage of ignition, but the process is slow. The front of the flame presents a light and dark plume shape, and the combustion zone shrinks continuously after weak expansion in the early time, and the explosion flame extinguishes itself at about 400 ms.

#### 3.2.2 Inerting effect of ultrafine Al(OH)_3_ on explosion flame propagation

For the samples with a mass concentration of 300 g/m^3^, the explosion flame front propagation process and velocity change curve under different inerting ratio conditions were tested to analyze the effect of different inerting ratios on the explosion flame propagation characteristics of high-purity aluminium dust and aluminium alloy polishing waste dust, the analysis results are as follows.

(1) Effect of different inerting ratios on the flame propagation characteristics of high-purity aluminum dust explosions.

**Fig. 10:**
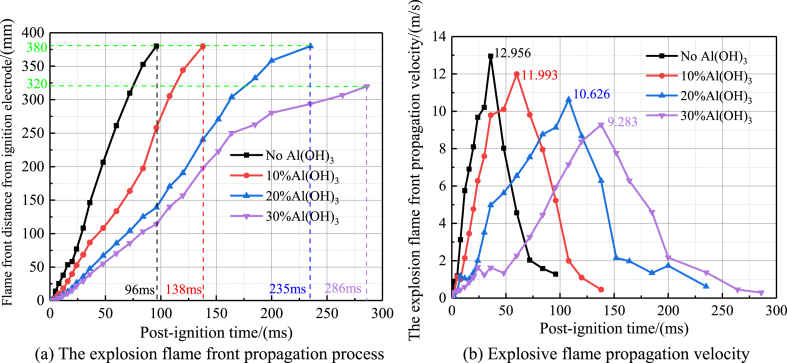
Propagation process and velocity curve of high-purity aluminum dust explosion flame front under different inerting ratios.

When the inerting ratio is 0% (no inerting agent added), 10%, 20% and 30%, the explosion flame front propagation process and the velocity change curve of the high-purity aluminium dust are shown in Fig. 10. As can be seen from Fig. 10(a), when ultrafine Al(OH)_3_ inerting agent is not added, after ignition 96 ms, the explosion flame front propagates to 380 mm above the electrode, when the inerting ratio is 10% and 20%, after ignition 138 ms and 235 ms, the explosion flame front can only propagate to 380 mm above the electrode, even inerting ratio of 30%, 286 ms after ignition, the explosion flame front can only propagate to 320 mm above the electrode. Therefore, as the inerting ratio increases, there is a significant delay in the propagation of the explosion flame front. As can be seen from Fig. 10(b), when no Al(OH)_3_ ultrafine inerting agent is added, the explosion flame propagation speed reaches a peak value of 12.96 m/s after ignition at 35 ms, and then the inerting ratio is gradually increased to 10%, 20% and 30%, after ignition at 60 ms, 110 ms and 140 ms, the corresponding peak explosion flame propagation speeds are 11.99 m/s, 10.63 m/s and 9.28 m/s, respectively. Therefore, as the inerting ratio increases, the explosion flame propagation speed continues to slow down and shows a trend of oscillation reduction.

(2) Effect of different inerting ratios on the flame propagation characteristics of aluminum alloy polishing waste dust explosions.

**Fig. 11:**
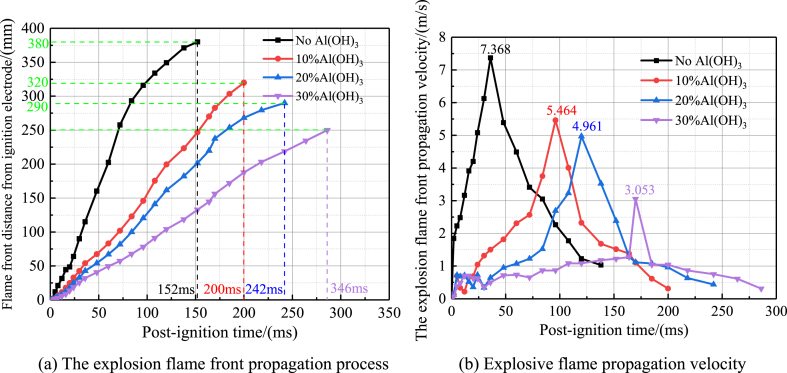
Propagation process and velocity curve of aluminum alloy polishing dust under different inerting ratio conditions.

When the inerting ratio is 0% (no inerting agent added), 10%, 20% and 30%, the explosion flame front propagation process and velocity change curve of high-purity aluminium dust are shown in Fig. 11. As can be seen from Fig. 11(a), when no Al (OH)_3_ ultrafine inerting agent is added, the explosion flame front propagates to 380 mm above the electrode after 152 ms of ignition, and when the inerting ratio is 10%, 20% and 30%, the explosion flame front propagates to 320 mm, 290 mm and 250 mm above the electrode after 200 ms, 242 ms and 346 ms of ignition, respectively. Therefore, an increase in the inerting ratio significantly delays the propagation of the explosion flame front. As can be seen from Fig. 11(b), when no Al(OH)_3_ ultrafine inerting agent is added, the explosion flame propagation speed reaches a peak value of 7.368 m/s after ignition at 35 ms, and then the inerting ratio is gradually increased to 10%, 20% and 30%, after ignition at 98 ms, 120 ms and 170 ms, the corresponding peak explosion flame propagation speeds are 5.464 m/s, 4.961 m/s and 3.053 m/s, respectively. Therefore, as the inerting ratio increases, the explosion flame propagation speed continues to slow down and shows a trend of oscillation reduction.

According to the analysis of the experimental results, the increase in the inerting ratio of the explosion flame propagation speed slowed down significantly, and show a trend of oscillation reduction, when the inerting ratio of 30%, can reduce the ignition sensitivity more significantly, the explosion flame after a relatively slow propagation, will also extinguish itself, to further improve the amount of Al(OH)_3_ powder applied, even under strong ignition conditions, did not occur in the flame sustained propagation phenomenon. Aluminum alloy polishing waste dust mixed with Al(OH)_3_ ultrafine dust can, on the one hand, reduce the local dust concentration of combustible dust, so that the risk of explosion becomes smaller. On the other hand, due to the small size of the inert powder, easy to suspend in the air, when the Al(OH)_3_ ultrafine powder is adsorbed on the surface of the aluminium alloy polishing waste dust particles, it can reduce their contact area with oxygen, interrupting the chain reaction in the combustion process. After the application of ultrafine Al(OH)_3_ powder, the discrete phenomenon of the burning zone of aluminium alloy polishing waste dust is obvious, the flame is difficult to form a continuous front, with the gradual melting of small particles of aluminium alloy polishing waste dust, decomposition, ultrafine Al(OH)_3_ powder absorbed most of its heat transferred by heat conduction and flame radiation, Only by residual heat can not maintain the preheating zone of combustible dust particles further decomposition, the large particles of combustible dust begin to fall under the action of gravity, the flame shows a disordered, discrete state, and the flame radiation intensity is greatly reduced.

## 4 Conclusion

Through the experimental test on the explosion propagation characteristics of high-purity aluminium dust and aluminium alloy polishing waste dust under different inerting ratios, the comparative analysis of the differences in the characteristics of explosion sensitivity, explosion propagation intensity and explosion flame propagation shape evolution, and the following conclusions are obtained.

(1) For aluminium alloy polishing waste dust, its combustion reaction activity, minimum ignition energy, lower explosion concentration and peak explosion flame propagation rate are significantly lower than those of high-purity aluminium dust of the same size, and according to the SEM image analysis of the experimental samples of different types of aluminium dust, the dust microstructure differences also have some influence on the explosion propagation characteristics. Therefore, in engineering practice, it is not appropriate to use the explosion parameters associated with high-purity aluminium dust as the basis for assessing the risk of combustion and explosion in aluminium alloy polishing operations.
(2) When the inerting ratio of Al(OH)_3_ ultrafine powder is 30%, the ignition energy of polishing waste dust can be inerted from 550 mJ to about 1 J, and the lower explosion limit is increased from 150 mg/m^3^ to 190 mg/m^3^, and the explosion risk is significantly reduced.
(3) The inerting ratio of ultrafine Al(OH)_3_ powder is 30%, high-purity aluminium powder explosion flame propagation velocity peak is still high to about 9.283 m/s, while flame propagation of polishing waste dust cloud is close to the critical state, for some aluminium alloy polishing site process, the explosion-proof measures have the potential of realistic feasibility, and can make the production waste to be resourceful reuse, to achieve efficient environmental protection and energy saving.
(4) The inhibition characteristic of the aluminum alloy polishing waste explosion by the addition of ultrafine Al(OH)_3_ inerting agent was explored in this article, taking into account the propagation law of the explosive flame in the pipeline. Yet, in a confined location, the explosion pressure is a critical metric for measuring the explosive strength and the suppression impact of the inerting agent. The current test apparatus will be improved in future studies, and the transient signal characteristics of the explosion pressure will be collected by placing pressure sensors in reasonable locations, revealing the influence of the explosion pressure propagation law of polishing aluminum alloy wastes with different concentrations and particle sizes by addition of ultrafine Al(OH)_3_ inerting agent.

## Author contribution statement

Chen Lv: Conceived and designed the experiments; Analyzed and interpreted the data; Wrote the paper.

Xinqun Wang: Conceived and designed the experiments; Contributed reagents, materials, analysis tools or data.

Sheng Xue: Analyzed and interpreted the data.

Xinxing Xia: Shuang Wang: Performed the experiments.

## Data availability statement

Data will be made available on request.

## Declaration of competing interest

No potential conflict of interest was reported by the authors.
